# Genotype and Phenotype Differences in CADASIL from an Asian Perspective

**DOI:** 10.3390/ijms231911506

**Published:** 2022-09-29

**Authors:** Yerim Kim, Jong Seok Bae, Ju-Young Lee, Hong Ki Song, Ju-Hun Lee, Minwoo Lee, Chulho Kim, Sang-Hwa Lee

**Affiliations:** 1 Department of Neurology, Kangdong Sacred Heart Hospital, Hallym University College of Medicine, Seoul 05355, Korea; 2 Department of Neurology, Hallym University Sacred Heart Hospital, Hallym University College of Medicine, Anyang 14068, Korea; 3 Department of Neurology, Chuncheon Sacred Heart Hospital, Hallym University College of Medicine, Chuncheon 24253, Korea

**Keywords:** CADASIL, mutation, NOTCH3 protein, stroke, cerebral infarction, intracranial hemorrhage

## Abstract

Cerebral autosomal dominant arteriopathy with subcortical infarcts and leukoencephalopathy (CADASIL) is a hereditary cerebral small-vessel disease caused by mutations in the NOTCH3 gene. Classical pathogenic mechanisms are associated with cysteine gain or loss, but recent studies suggest that cysteine-sparing mutations might have a potential role as a pathogen. In comparison with CADASIL patients in Western countries, there are several differences in Asian patients: (1) prevalent locus of NOTCH3 mutations (exons 2–6 [particularly exon 4] vs. exon 11), (2) age at symptom onset, (3) prevalence of cerebral microbleeds and hemorrhagic stroke, (4) clinical symptoms, and (5) severity of white matter hyperintensities and typical involvement of the anterior temporal pole in magnetic resonance imaging. Both ethnicity and founder effects contribute to these differences in the clinical NOTCH3 spectrum in different cohorts. More functional investigations from diverse races are needed to clarify unknown but novel variants of NOTCH3 mutations. This review may broaden the spectrum of NOTCH3 variants from an Asian perspective and draw attention to the hidden pathogenic roles of NOTCH3 variants.

## 1. Introduction

**Figure 1:**
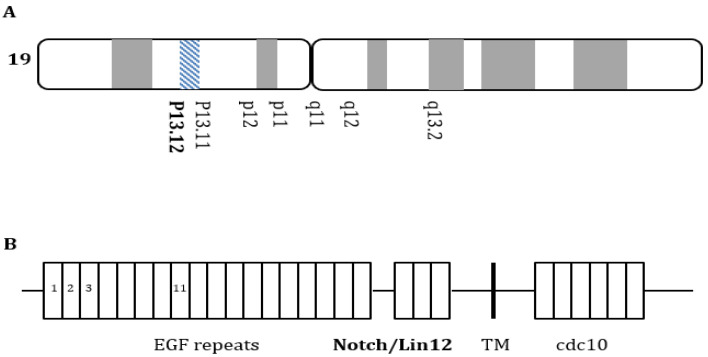
NOTCH3 gene mutations. (**A**) NOTCH3 gene located on chromosome 19p13.12; (**B**) NOTCH3 gene mutations are located within the extracellular domain that encodes the epidermal growth factor-like repeats, three Lin-NOTCH repeats, and one transmembrane region.

Cerebral autosomal dominant arteriopathy with subcortical infarcts and leukoencephalopathy (CADASIL) is a hereditary cerebral small-vessel disease caused by mutations in the NOTCH3 gene located on chromosome 19p13.12 [1]. This gene contains 33 exons encoding the NOTCH3 protein, and NOTCH3 contains three domains: (1) a large extracellular domain (ECD) with 34 epidermal growth factor-like repeats (EGFrs), (2) a transmembrane domain, and (3) an intracellular domain (Figure 1) [2]. Each EGFr contains six cysteine residues that form three pairs of disulfide bonds to maintain the normal NOTCH3 protein’s tertiary structure [1,2,3]. The classical pathogenic mechanisms are associated with cysteine gain or loss, enhanced oligomer formation, and ECD aggregation [1].

The major clinical symptoms of CADASIL are recurrent small subcortical infarctions, migraines, psychiatric disturbances, seizures, and cognitive decline [4]. Typical magnetic resonance imaging (MRI) is characterized by small lacunar infarcts and severe white matter hyperintensities (WMHs), mostly in periventricular lesions, with involvement of the anterior temporal pole (ATP) and external capsule (EC) [1,5] Additionally, granular osmiophilic material (GOM) is typically detected in the extracellular space [5].

Interestingly, most of the pathogenic mutations are presented in exons 2–24, but recent studies have found that mutations outside the EGFr coding region (exons 25–33) may be pathogenic in CADASIL [6]. Contrary to the typical mechanism of CADASIL, cysteine-sparing NOTCH3 mutations as well as homozygous NOTCH3 mutations have been reported [3,7]. In addition, NOTCH3 mutations are not detected in some CADASIL patients, [8] and typical MRI findings or pathological findings, such as the presence of GOM, have not been observed [1,4,7]. Although there is still debate, these phenomena seem to be associated with specific genotypes, which may affect clinical differences in ancestry or founder effect.

We reviewed the clinical differences in CADASIL features according to ancestry, considering genetic aspects, differences between cysteine-related and cysteine-sparing mutations, and region-specific founder effects.

## 2. Genotype and Phenotype Differences between Asians and Patients of European Ancestry

### 2.1. Mutations in Different Exons

In comparison with CADASIL patients in Western countries, there are several differences in Asian patients. First, in most patients of European ancestry, NOTCH3 mutations are observed in exons 2–6 (60–80%), particularly in exon 4 (50–75%) [8,9,10] (Table 1). Among them, NOTCH3 mutations in patients in the United Kingdom, France, and Germany most commonly occur in exon 4, followed by exons 3–6 [9,10,11,12]. However, in a Dutch study, exons 4 and 11 were the most frequent areas [13]. In Italian patients, mutations in exon 4 accounted for only 20.6% [14]. In Asian populations, NOTCH3 mutations were more frequently detected in exon 11 (40–85%) and exon 4 (20–40%) in Korea [7], mainland China [15], and Taiwan [16]. In particular, R544C in exon 11 accounts for the most common mutation in NOTCH3 in the South Korean population (Jeju Island) [17,18]. However, mutations in exon 11 are rare in the Japanese population. In a Japanese study that included 70 CADASIL patients, R544C was not detected [19]. Recent studies have demonstrated that patients with mutations in EGFrs 1–6 had a 12-year early onset of stroke, greater disease severity, and higher WMH than those with mutations in EGFrs 7–34 [20,21]. A previous study investigating 76 cysteine-related NOTCH3 variants in 485 CADASIL patients reported that the EGFr 1–6 phenotype was correlated with greater disease severity [21]. After controlling for cardiovascular risk factors, patients with mutations in EGFrs 1–6 had a 2.05-fold risk of earlier stroke onset and 2.70-fold risk of encephalopathy compared to those with mutations in EGFrs 7–34. In particular, mutations in EGFrs 10–17 were associated with a lower stroke risk [21,22].

Of note, as described above, NOTCH3 mutations in Europeans accumulated in EGFrs 1–6 at 71.8% [20]. These results suggest that the classical phenotypes of CADASIL might be different in different ethnic groups.

### 2.2. Age at Symptom Onset

Asian CADASIL patients are older at symptom onset than Western CADASIL patients [1,16,17,23]. In Asian reports, the mean age at symptom onset was 54.1 ± 12.5 (Taiwan), 42.7 ± 9.1 (China), and 53.4 ± 9.9 (South Korea) years [7]. In Western studies, the mean age at symptom onset was 35.9 ± 14.6 (UK) [10], 33.6 ± 14.1 (UK) [24], and 48.5 ± 17.1 (Italy) [14] years. We cannot confirm whether this is a racial difference; a recent study demonstrated that the EGFr 1–6 phenotype has been correlated with earlier stroke onset than variants in other EGFr domains [21]. Since most Western CADASIL mutations were detected in exons 2–6 [10], this may affect earlier symptom onset.

### 2.3. Cerebral Microbleeds and Hemorrhagic Stroke

The main clinical symptom of CADASIL is recurrent ischemic stroke (IS) with severe white matter changes. However, Asians have a higher incidence of cerebral microbleeds (CMBs) and hemorrhagic stroke (HS) [10,23]. Recent studies from Korea and Taiwan reported that 25% of CADASIL patients had intracerebral hemorrhage (ICH). ICH development seems to be closely related to CMBs; this has been described in a Taiwanese cohort (87.5%) [16] and in Jeju Island in South Korea (73.3%) [17]. In contrast, ICH development was only 1% in Finland [25] and CMBs were present in 31% of CADASIL patients in the Netherlands [26]. The frequency of CMBs in HS patients was higher than that in IS patients [27]. A previous meta-analysis demonstrated that associations between CMBs and ICH were more significant in Asian (OR, 10.43) than in Western cohorts [28]. These ethnic differences may affect CADASIL stroke phenotypes.

### 2.4. Differences in Clinical Features

CADASIL in Asian patients has a rarer manifestation of migraines, seizures, psychiatric disorders, and dementia. Migraines are the most common early symptom, with an incidence of 22–77% [29]. Migraines usually start in the second decade and appear in 90% of patients before 40 years of age [29]. Although the exact pathomechanisms are not yet elucidated, migraine attacks appear 10 years earlier than cerebral ischemic attacks, and these headaches do not seem to be caused by stroke. Interestingly, Asian CADASIL studies have found a lower prevalence of migraines. In Asian cohorts, headache prevalence was 2.5% in Korea [23], 4.8% in Taiwan [30], 8.1% in mainland China [31], and 33% in Japan [19]. In contrast, the prevalence was 54.2% in British [10] and 40% in American patients [31].

The second most frequent symptom is cognitive decline, which accounts for 60% of CADASIL patients [29]. The prevalence of dementia varied according to the ancestry: Korea (15%, 18.5%, and 43%) [17,22,32], Taiwan (4.8%, and 41.1%) [16,30], mainland China (11.3%) [31], Japan (31%) [19], the UK (2.1% and 16%) [10,24], and the United States (5.7% and 15.6%) [33,34]. Given these distributions, it is thought that this difference is determined by the presence of a particular gene mutation rather than by the ancestry. For example, cognitive decline was more common in the R544C carriers than in the R75P carriers [32]. In addition, psychiatric symptoms (20–41%) and seizures (5–10%) occur in patients with CADASIL [29]. There seems to be no specific racial differences reported for these symptoms.

**Table 1:** Comparison of the different spectrum of NOTCH3, MRI features, and clinical presentations of CADASIL.

Study	Ancestry	Number of Patients	Age at Onset (±SD)	Mutation of NOTCH3	MRI Features	Clinical Presentations				
						Stroke	Cognitive Impairment	Psychiatric Syndrome	Headaches	Seizures
Adib-Samil et al., 2010 [24]	UK	200	33.6 ± 14.1	Exons 2–6: 91.9% (R544C: 0%)	NS	51.5%	16%	37.5%	75%	10.5% (encephalopathy)
Markus et al., 2002 [10]	British	48	35.9 ± 14.6	Exons 2–6: 93.8%	AT: 89% EC: 93%	IS: 29.2% ICH: NS	2.1%	8.3%	54.2%	4.2%
Ospina et al., 2020 [34]	USA	90	Age at first evaluation, median (IQR) 36 (24)	Exons 4 (R141C): 23.3% Exon 14 (R1031C): 65.5%	NS	Stroke: 35.6%	15.6%	33.3%	43.3%	NS
Desmond et al., 1999 [33]	USA	105	36.7 ± 12.9	Not specified	NS	IS: 42.9%	5.7%	8.6%	40%	2.9%
Dotti et al., 2005 [35]	Italian	28	NS	Exons 2–6: 46.4% Exon 11: 21.4%	NS	NS	NS	NS	NS	NS
Bianchi et al., 2015 [14]	Italian	229	48.5 ± 17.1	Exons 2–6: 36.7% (R544C: 0%)	NS	59%	38%	48%	42%	8%
Mönkäre et al., 2022 [25]	Finnish	294	50 ± 13.7	Exon 2–6: 74.8% (R133C: 68%)	NS	IS or TIA: 42% ^a^ ICH: 1% ^a^	31% ^a^	11% ^a^	34% ^a^	4% ^a^
Lee et al., 2009 [30]	Han Chinese in Taiwan	21	48.6 ± 13.8	Exons 2–6: 28.6% Exon 11 (R544C): 47.6%	AT: 42% EC: 95.2%	IS: 52.4% ICH: 23.8%	4.8%	9.5%	4.8%	4.8%
Liao et al., 2015 [16]	Taiwan	95	54.1 ± 12.5	Exons 2–6: 20% Exon 11 (R544C: 70.5%)	AT: 44.8% EC: 85.4% (AT: 28.4% EC: 83.6%)	76.8% (73.4%)	41.1% (48.1%)	15.2% (15.2%)	2.7% (3.8%)	NS
Liu et al., 2015 [31]	Chinese mainland	62	39.7 ± 8.03	Exon 4: 59.6% Exon 3: 22.8% Exon 11: 3.5%	AT: 63.5% * EC: 69.2% *	IS or TIA: 75.8%	11.3%	3.2%	8.1%	NS
Choi et al., 2006 [17]	Korean	20	57.2 ± 10.2	Exons 2–6 (R75P): 10% Exon 11: 85% (R544C: 75%)	AT: 20% EC: 90%	IS: 55% ICH: 25%	15%	0	10%	0
Choi et al., 2013 [18]	Korean	73	62.7 ± 11.1	Exons 2–6 (R75P): 2.7% Exon 11: 95.9% (R544C: 90.3%)	NS	IS: 42.5% ICH: 12.3%	NS	NS	NS	NS
Kim et al., 2006 [22]	Korean	27	47.7	Exons 2–6: 77.8% (R75P: 55.6%) Exon 11: 22.2%	AT: 23.0% EC: 53.8%	IS: 40.7% ICH: 33.3%	18.5%	Depression: 14.8%	3.7%	0
Kim et al., 2019 [23]	Korean	34	52.5 ± 9.5	Exons 2–6: 41.2% (R75P/Q *: 17.6%) Exon 11: 26.5%	AT: 50% EC: 55.9%	IS: 44.1% ICH: 17.6%	NS	0	2.5%	0
Min et al., 2022 [32]	Korean	142	51.2 ± 10	Exon 2–6: 54.93% Exon 11: 34.5% (R544C: 20.4%)	AT: 61.2% EC: 76.7%	IS or TIA: 61.3% ICH: 6.3%	43%	20.4%	40.1%	0
Ueda et al., 2015 [19]	Japanese	70	R75P: 53.6 ± 6.9 other mutations: 44.2 ± 12.0	Exon 3: 21% Exon 4: 69%	AT: 70.6% **†** EC: 76.5% **†**	IS or TIA: 69%	31%	20%	33%	NS

Abbreviations: SD, standard deviation; AT, anterior temporal; EC, external capsular; IS, ischemic; ICH, intracerebral hemorrhage; NS, not specified. * Frequency was calculated for 52 patients with CADASIL scores. **†** Frequency was calculated for 51 CADASIL patients using available MRI data. ^a^ These were analyzed only in 200 individuals with the R133C mutation.

### 2.5. Imaging Differences

**Figure 2:**
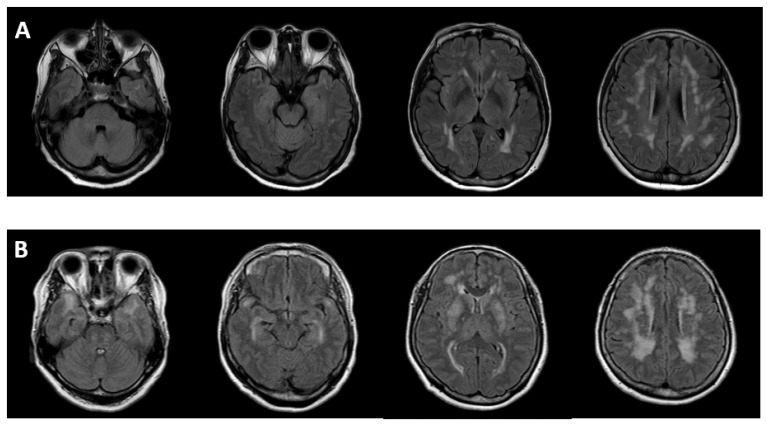
Anterior temporal pole involvement according to cysteine-sparing or cysteine-related mutations. (**A**) cysteine-sparing (R75P mutation in exon 3); (**B**) cysteine-related (C542R mutation in exon 11).

Asian patients have a lower prevalence of involvement of ATP and EC with WMH than those patients of European ancestry. In Western studies, the prevalence of involvement of ATP and EC was 89% and 93%, respectively [10]. The specificity was much higher for ATP than for EC (100% vs. 45%). In contrast, the prevalence of ATP involvement was only 20% [17] and 23% [22] in Korea. In 112 patients included in a Taiwanese study, the overall prevalence of ATP involvement was 44.5%, while the prevalence of ATP involvement in only R544C patients was only 28.4%. This means that other regions of mutations account for 82.8% of cases of ATP involvement [16]. This finding suggests that there is an important correlation between the location of mutations and typical WMH. Furthermore, in a British study, NOTCH3 mutations were clustered in exons 2–6 in 93.8% of cases, while R544C mutations were never observed (0%) [10]. On the contrary, R544C in exon 11 was the most common region of NOTCH3 mutation in an Asian cohort. In a Korean study in Jeju Island, the overall prevalence of ATP involvement was only 20%, and R544C mutations accounted for three quarters (75%) of the total population [17]. Another Korean study including 42 subjects from 9 unrelated families found R75P mutations in 4 families. While ATP regions were observed in 75% (6/8) of subjects without R75P mutations, these were detected in none of the patients with R75P mutations [22] (Figure 2).

## 3. Cysteine-Sparing and Cysteine-Related Mutations

Although there is still debate, accumulating evidence suggests that clinical and imaging findings vary across different regions [3]. The major pathogenicity of NOTCH3 mutations results in the loss or gain of one cysteine residue, leading to odd numbers [1]. At first, the possible pathogenicity of cysteine-sparing mutations was controversial. However, recent evidence suggests that several cysteine-sparing mutations (R61W, R75P, D80G, and R213K) may be potentially pathogenic because they have typical clinical CADASIL features and extensive WMH, no other potential pathogenic mutations, minor allele frequency according to Exome Aggregation Consortium <0.1%, and GOM deposits in skin biopsy [1]. Hu et al. reported that cysteine-sparing variants showed later symptom onset than cysteine-related variants (age 51 ± 7 vs. 45 ± 9 years). Although the prevalence of ATP involvement was not significantly different between the two groups, WMH rating scales indicated that the severity of ATP hyperintensities was lower in subjects with cysteine-sparing variants [4]. Similarly, although it is small, the proportion of ATP involvement in R75P/R75Q was only 16.7% (cysteine-sparing), whereas that in cysteine-related regions (C123Y, R169C, C185Y, and C212G) was 100% in a previous Korean study [23]. A representative cysteine-sparing mutation of R75P was commonly reported in Korean and Japanese patients, but it was not found in Chinese patients and patients of European ancestry [19]. This mutation is associated with arginine, and several mutations at arginine residues have been reported to be functionally related to NOTCH3 [22]. For example, arginine 184 is the most frequently mutated region in JAG1, which encodes a NOTCH ligand protein containing 16 EGFr [36]. It is important to note why R544C in exon 11, which is common in Koreans and Taiwanese patients, is an R544C cysteine-related mutation, but the disease severity is weak. We cannot explain the exact pathomechanisms, but as mentioned above, recent studies identified that patients with mutations in EGFrs 1–6 had a significantly earlier onset of stroke, greater disease severity, and higher WMH than those with mutations in EGFrs 7–34 [20,21]. Interestingly, the R544C mutation occurs at a single amino acid bordered between the EGFr-13 and -14 domains, in contrast to other cysteine-related mutations residing within an EGFr domain [4,5].

## 4. Founder Effect

**Figure 3:**
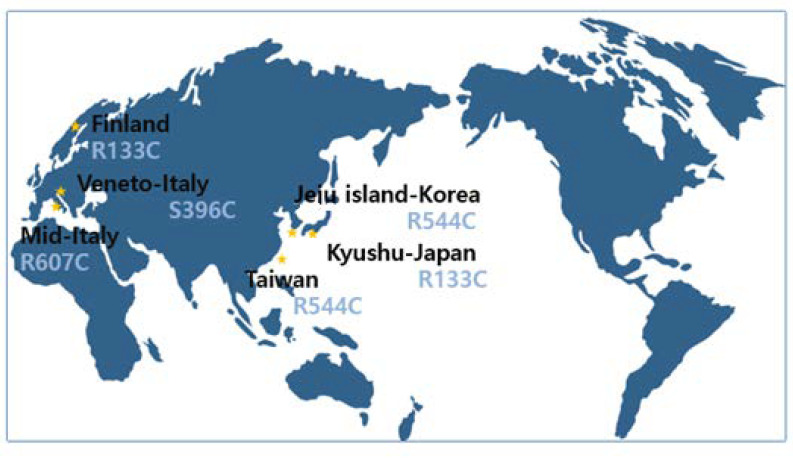
“Founder effect” reported in many countries: Taiwan (R544C) [29], Jeju island in Korea (R544C) [17,18], Finland (R133C) [37], the Kyushu area in Japan (R133C) [19], mid-Italy (R607C) [35] and the Veneto region in Italy (S396C) [38]. * This figure was modified from [23].

In addition to ethnic differences, the founder effect contributes to different characteristics of the NOTCH3 spectrum in CADASIL. The founder effect has been detected in small, isolated geographical areas, such as Taiwan (R544C) [29], Jeju Island in Korea (R544C) [17,18], Finland (R133C) [37], the Kyushu area in Japan (R133C) [19], mid-Italy (R607C) [35], and the Veneto region in Italy (S396C) [38]. (Figure 3) This phenomenon occurs when a small group of individuals is isolated from a larger population, leading to a reduction in genetic variation. Broadly, R75P mutations have been reported in Korean and Japanese CADASIL patients but not in Chinese or Western populations. Therefore, some researchers have suggested that R75P might be another cause of the founder effect in Far East Asian populations [19].

## 5. Conclusions

Accumulating evidence suggests clinical associations between the genotype and phenotype profiles of CADASIL. Unlike in westernized countries, typical CADASIL imaging markers, such as ATP or EC involvement, may not be detected—at least in some Asian populations. In addition, physicians have to pay attention to the increasing HS risk in managing Asian individuals. Both ancestry and founder effects contributed to these differences in the NOTCH3 spectrum in different populations. However, the pathogenic role of cysteine-sparing mutations has not been elucidated. Further studies in diverse ancestries are necessary to clarify unknown but novel variants of NOTCH3 mutations. This review may broaden the spectrum of NOTCH3 variants from an Asian perspective and draw attention to hidden pathogens of NOTCH3 variants.

## Data Availability

Not applicable.
